# QuestNova: inovação na avaliação do consumo alimentar segundo o processamento industrial

**DOI:** 10.11606/s1518-8787.2024058006307

**Published:** 2024-10-22

**Authors:** Maria Laura da Costa Louzada, Thays Nascimento Souza, Evelyn Frade, Kamila Tiemann Gabe, Gustavo Akinaga Patricio

**Affiliations:** I Universidade de São Paulo. Faculdade de Saúde Pública. Núcleo de Pesquisas Epidemiológicas em Nutrição e Saúde. São Paulo, SP, Brasil - Av. Dr. Arnaldo, 715 01246-904; II Universidade de São Paulo. Faculdade de Saúde Pública. São Paulo, SP, Brasil

**Keywords:** Consumo Alimentar, Processamento de Alimentos, Software, Coleta de Dados, Alimentos Ultraprocessados, Internet, Inquéritos Nutricionais, Inquéritos e Questionários, Inquéritos sobre Dietas, Pesquisadores

## Abstract

O objetivo deste comentário é descrever as características, o desenvolvimento e as funcionalidades da plataforma de coleta de dados de consumo alimentar QuestNova. A plataforma foi desenvolvida por dois especialistas em tecnologia da informação, com suporte de uma equipe do Núcleo de Pesquisas Epidemiológicas em Nutrição e Saúde da Universidade de São Paulo (Nupens/USP). O processo de desenvolvimento ocorreu em etapas, com todas as funcionalidades de cada passo sendo minuciosamente testadas por múltiplos membros da equipe antes de avançar para o próximo. A QuestNova é uma plataforma online gratuita, que disponibiliza três instrumentos autoaplicáveis para avaliação do consumo alimentar, com base na classificação Nova: Screener-Nova, QFA-Nova e R24h-Nova. Na plataforma, o pesquisador pode selecionar o instrumento de interesse e enviá-lo por meio de um link aos participantes de sua pesquisa, que o responderão de forma autônoma, dispensando a presença de um entrevistador. Bancos de dados contendo indicadores relevantes para a avaliação da alimentação segundo o nível de processamento são gerados automaticamente a partir das respostas. Um aspecto crucial da QuestNova é o seu compromisso com a confidencialidade e a segurança dos dados dos participantes. Nenhuma informação é armazenada internamente na plataforma; pelo contrário, os dados são transmitidos diretamente para uma conta no Google Drive fornecida pelo próprio pesquisador. A QuestNova democratiza o acesso a instrumentos de pesquisa inovadores, potencializando estudos sobre o impacto do processamento de alimentos na saúde brasileira. Atualizações futuras podem ampliar sua utilidade.

## INTRODUÇÃO

 Em 2010, pesquisadores do Núcleo de Pesquisas Epidemiológicas em Nutrição e Saúde da Universidade de São Paulo (Nupens/USP) propuseram uma nova classificação de alimentos, denominada Nova ^ Monteiro *et al.* , 2010 ^ . Esta classificação agrupa os alimentos em categorias de acordo com as características do processamento industrial: (1) alimentos *in natura* ou minimamente processados, (2) ingredientes culinários processados, (3) alimentos processados e (4) alimentos ultraprocessados ^ Monteiro *et al.* , 2019 ^ . 

 A classificação Nova provocou uma ruptura paradigmática na ciência da nutrição, passando a ser incorporada em um crescente número de estudos epidemiológicos. Entretanto, um desafio comum da aplicação da Nova nesses estudos foi o uso de dados de consumo alimentar coletados a partir de instrumentos incapazes de captar diferenças de processamento industrial. Neles, por exemplo, era possível que todas as sopas de legumes fossem consideradas similares, independentemente de serem caseiras, enlatadas ou desidratadas industrialmente. Dessa forma, poderiam induzir a vieses na estimação do consumo de grupos de alimentos segundo a classificação Nova e, consequentemente, na identificação dos seus determinantes e consequências ^ Touvier *et al.* , 2023 ^^,^^Martinez-Steele *et al.* , 2023 ^ . 

 Para superar parte desta limitação, uma equipe do Nupens/USP desenvolveu e validou três novos instrumentos eletrônicos autoaplicáveis para avaliar o consumo de alimentos segundo a classificação: o rastreador do consumo de alimentos *in natura* ou minimamente processados integrais e de origem vegetal e de alimentos ultraprocessados ( *Screener* -Nova) ^ Costa *et al.* , 2021a ^^,^^ Costa *et al.* , 2023 ^ ; o recordatório alimentar de 24h-Nova (R24h-Nova) ^ Neri *et al.* , 2023 ^ ; e o Questionário de Frequência Alimentar-Nova (QFA-Nova) ^ Frade *et al.* , 2024 ^ . 

Apesar do crescente interesse dos pesquisadores por esses novos instrumentos, ainda não existia uma plataforma online que os disponibilizasse. Isso exigia que cada pesquisador realizasse sua própria programação, o que representava obstáculos consideráveis – como tempo, custos e recursos tecnológicos –, tornando os instrumentos inacessíveis para uma parcela significativa da comunidade científica. A plataforma QuestNova surgiu como resposta a essa necessidade premente. O objetivo deste comentário é descrever as características, o desenvolvimento e as funcionalidades dessa plataforma.

## PLATAFORMA QUESTNOVA: CARACTERIZAÇÃO E DESENVOLVIMENTO

A QuestNova é uma plataforma online que disponibiliza gratuitamente os três instrumentos para coleta de dados de consumo alimentar para pesquisadores: o Screener-Nova, o QFA-Nova e o R24h-Nova. Na plataforma, o pesquisador pode selecionar o instrumento de interesse e enviá-lo por meio de um link aos participantes de sua pesquisa, que o responderão de forma autônoma, dispensando a presença de um entrevistador. Bancos de dados contendo indicadores relevantes para a avaliação da alimentação segundo o nível de processamento são gerados automaticamente a partir das respostas, o que poupa pesquisadores das etapas de processamento de dados brutos e de cálculo de indicadores.

 A plataforma foi desenvolvida por dois especialistas em tecnologia da informação, com suporte de uma equipe do Nupens/USP, que é composta, em parte, por pesquisadores que também participaram do desenvolvimento dos instrumentos de avaliação do consumo alimentar. O processo de desenvolvimento ocorreu em etapas, com todas as funcionalidades de cada passo sendo minuciosamente testadas por múltiplos membros da equipe antes de avançar para o próximo. A ferramenta se destaca por sua interface intuitiva e design responsivo, que permite o acesso tanto via computador quanto por celular (IOS ou Android) e tablet. Ela pode ser acessada em https://questnova.com.br/ . 

Um aspecto crucial da QuestNova é o seu compromisso com a confidencialidade e segurança dos dados dos participantes. Nenhuma informação é armazenada internamente na plataforma; pelo contrário, os dados são transmitidos diretamente para uma conta no Google Drive fornecida pelo próprio pesquisador, garantindo assim a privacidade das informações coletadas.

## INSTRUMENTOS DE COLETA DE DADOS DE CONSUMO ALIMENTAR E INDICADORES DE AVALIAÇÃO DE QUALIDADE DA DIETA

Os três instrumentos foram desenvolvidos com base nos dados de consumo alimentar individual da população brasileira descritos nas Pesquisas de Orçamentos Familiares.

### Screener-Nova

 O Screener-Nova é um instrumento curto de avaliação do consumo de alimentos *in natura* ou minimamente processados integrais e de origem vegetal e de alimentos ultraprocessados. O respondente deve selecionar os alimentos consumidos no dia anterior em uma lista de 33 itens do primeiro grupo e 23 do segundo (sem informar quantidades). O tempo médio de preenchimento do instrumento é de apenas 2 minutos. As respostas resultam em dois escores separados para cada conjunto de alimentos, os quais são calculados por meio da soma simples de um ponto para cada alimento consumido e disponibilizados no banco de dados da QuestNova. Estudo de validação revelou que cada um dos escores foi fortemente associado com a participação de energia do correspondente grupo, obtida a partir de um R24h ^ Costa *et al.* , 2021a ^^,^^ Costa *et al.* , 2023 ^ . 

### R24h-Nova

 O R24h-Nova é um instrumento fechado que registra o consumo de todos os alimentos do dia anterior. O instrumento tem 57 perguntas-chave do tipo “sim” ou “não”, que indagam sobre o consumo de alimentos-chave. Respostas afirmativas desencadeiam uma série de perguntas adicionais sobre tipo e quantidade consumida, método de preparo, itens de adição e detalhes para distinguir os alimentos quanto ao grau de processamento. O preenchimento do instrumento leva cerca de 15 minutos. O estudo de validação demonstrou uma correlação de moderada a boa entre as estimativas médias obtidas pelo R24h-Nova e aquelas obtidas por um R24h conduzido por um entrevistador, bem como uma concordância de substancial a quase perfeita para a capacidade do R24h-Nova em classificar os indivíduos em quantis da participação de energia, segundo os grupos da Nova ^ Neri *et al.* , 2023 ^ . Após o estudo de validação, foram realizados ajustes no instrumento para melhorar a sua performance na estimativa de itens que apresentaram menores concordâncias (por exemplo, melhora na pergunta sobre adição de azeite de oliva aos alimentos prontos). 

### QFA-Nova

 O QFA-Nova é um instrumento que avalia o consumo alimentar habitual nos últimos 12 meses. Composto por uma lista de 95 alimentos classificados de acordo com suas características de processamento industrial, o questionário investiga a frequência e a quantidade usual (porções) de consumo de cada item, requerendo cerca de 20 minutos para ser completado. Estudo de validação descreveu que as estimativas médias de participação calórica dos grupos da Nova obtidas pelo QFA-Nova foram moderadamente correlacionadas com aquelas obtidas da média de dois R24h, também foi observada concordância substancial na classificação de indivíduos em quantis da participação de energia de todos os grupos da Nova ^ Frade *et al.* , 2024 ^ . Levando em conta esses resultados, foram feitos pequenos ajustes no instrumento, a fim de melhorar a estimativa de grupos de alimentos com correlações moderadas (por exemplo, ajuste na porção de referência e no número de frutas perguntadas no questionário). 

 Nos bancos de dados gerados pela QuestNova para o QFA-Nova e para R24-Nova são disponibilizados, para cada indivíduo, a energia total consumida e os indicadores de consumo (em gramas, em energia e em percentuais de participação) de cada um dos grupos e subgrupos da classificação Nova. Para esses cálculos, são utilizadas as receitas e os dados de composição nutricional da Tabela Brasileira de Composição de Alimentos 7.0 (TBCA 7.0) ^Brazilian Table of Food Composition (TBCA), 2023^ ou, no caso excepcional de ausência de informações, em outras fontes – como a tabela da USDA ^US Department of Agriculture, 2004^ . 

## PASSO-A-PASSO PARA UTILIZAÇÃO DA PLATAFORMA QUESTNOVA

Abaixo, apresentamos um passo-a-passo simplificado com os procedimentos necessários para utilização da plataforma QuestNova pelos pesquisadores (Figura 1). Um manual completo e detalhado está disponível na própria plataforma.

Cadastro do pesquisador:

- O pesquisador deve se cadastrar na plataforma, fornecendo informações pessoais e uma breve descrição do objetivo de uso.
- É necessário que o e-mail cadastrado esteja vinculado a uma conta do Google Drive para o salvamento e sincronização dos dados.

Associação com o Google Drive:

- Após o cadastro, o pesquisador deve associar sua conta na plataforma QuestNova (em um local indicado na própria interface) com sua conta do Google Drive.
- Essa etapa é fundamental para que os dados coletados sejam salvos e sincronizados no Google Drive do pesquisador.

Registro da pesquisa:

- Com o cadastro e a associação do Google Drive concluídos, o pesquisador pode registrar sua pesquisa na plataforma.
- O pesquisador deve preencher informações como título da pesquisa, objetivo e estimativa do número de participantes, além de escolher o instrumento que deseja utilizar.
- É possível registrar quantas pesquisas forem desejadas.

Envio do link da pesquisa aos participantes:

- Após o registro da pesquisa, um link único é gerado, o qual deve ser enviado pelo pesquisador aos participantes de seu estudo, para que respondam ao instrumento.
- Os participantes devem inserir um e-mail válido e sua data de nascimento antes de responderem ao instrumento de avaliação do consumo alimentar. Estes dados, em conjunto, são a chave-primária para identificação dos participantes e, portanto, para que o pesquisador consiga juntar a resposta do participante às demais bases de dados de sua pesquisa

Acesso ao banco de dados:

- Os dados coletados são sincronizados no Google Drive do pesquisador.
- Uma pasta nomeada QuestNova é gerada, contendo o banco de dados da pesquisa em formato Google Sheets.
- O banco de dados inclui informações sobre o consumo alimentar dos participantes de acordo com a classificação Nova.

## CONSIDERAÇÕES FINAIS

Este manuscrito apresentou a plataforma QuestNova, que representa uma inovação tecnológica para a avaliação do consumo alimentar conforme proposto pela classificação de alimentos Nova. A plataforma simplifica a coleta e a análise de dados, poupando tempo e recursos tecnológicos dos pesquisadores.

 O R24h-Nova e o Screener-Nova são aplicados periodicamente no Estudo Nutrinet Brasil ^ Werneck *et al.* , 2024 ^^-^^ Gabe *et al.* , 2023 ^ . Alguns exemplos de estudo já realizados incluem um que mostrou uma associação entre a participação calórica de alimentos ultraprocessados (estimada pelo R24h-Nova) e o risco de depressão ^ Werneck *et al.* , 2024 ^ e outro que descreveu a relação entre os escores de alimentos ultraprocessados e de alimentos *in natura* ou minimamente processados (estimados pelo Screener-Nova) e o ganho de peso excessivo ^ Santos *et al.* , 2023 ^ . Além disso, uma versão preliminar do Screener-Nova foi incorporada no Vigitel 2018, na Pesquisa Nacional de Saúde 2019 e na Pesquisa Nacional de Saúde do Escolar 2019 ^ Costa *et al.* , 2021b ^^,^^ Costa *et al.* , 2022 ^ . 

A disponibilização da QuestNova democratiza o acesso a esses instrumentos, potencializando estudos brasileiros sobre o impacto do processamento de alimentos na saúde. Além disso, a QuestNova pode servir como modelo para outros países desenvolverem plataformas similares, que incorporem instrumentos que avaliem o consumo alimentar segundo a Nova. Futuras atualizações podem incluir novos instrumentos ou indicadores de qualidade da alimentação, fortalecendo ainda mais sua utilidade e relevância para a epidemiologia nutricional.
