# Supplementary material for: QuestNova: inovação na avaliação do consumo alimentar segundo o processamento industrial
Source: Rev Saude Publica. 2024 Oct 22;58:38. [Article in Portuguese] doi: 10.11606/s1518-8787.2024058006307 (PMC11584175; doi:10.11606/s1518-8787.2024058006307)
Supplement: Translation

# Translation: QuestNova: innovation in food consumption assessment according to industrial processing

**Published:** 2024-10-22

**Authors:** Maria Laura da Costa Louzada, Thays Nascimento Souza, Evelyn Frade, Kamila Tiemann Gabe, Gustavo Akinaga Patricio

**Affiliations:** I Universidade de São Paulo. Faculdade de Saúde Pública. Núcleo de Pesquisas Epidemiológicas em Nutrição e Saúde. São Paulo, SP, Brasil - Av. Dr. Arnaldo, 715 01246-904; II Universidade de São Paulo. Faculdade de Saúde Pública. São Paulo, SP, Brasil

**Keywords:** Food intake, Food Processing, Software, Data collection, Ultra-processed foods, Internet, Nutritional Surveys, Surveys and Questionnaires, Diet Surveys, Researchers

## Abstract

The objective of this commentary is to describe the characteristics, development and functionalities of the food intake data collection platform QuestNova. The platform was developed by two information technology specialists, with the support of a team from Nupens/USP. The development process took place in stages, with all the functionalities of each step being thoroughly tested by multiple team members before moving on to the next. QuestNova is a free online platform that offers three self-administered instruments for assessing food intake, based on the Nova classification: Screener-Nova, QFA-Nova and R24h-Nova. On the platform, the researcher can select the instrument of interest and send it via a link to the participants in their study, who will answer it autonomously, without the presence of an interviewer. Databases containing relevant indicators for evaluating food according to the level of processing are automatically generated from the responses. A crucial aspect of QuestNova is its commitment to the confidentiality and safety of participant data. No information is stored internally on the platform; on the contrary, data is transmitted directly to a Google Drive account provided by the researcher themselves. QuestNova democratizes access to innovative research tools, boosting studies on the impact of food processing on Brazilian health. Future updates may extend its usefulness.

## INTRODUCTION

 In 2010, researchers from the *Núcleo de Pesquisa Epidemiológicas em Nutrição e Saúde* of the Universidade de São Paulo (Nupens/USP – Nucleus of Epidemiological Research on Nutrition and Health at the University of São Paulo) proposed a new food classification, called Nova ^ Monteiro *et al.* , 2010 ^ . This classification groups foods into categories according to the characteristics of industrial processing: (1) *natural* or minimally processed food, (2) processed culinary ingredients, (3) processed food, and (4) ultra-processed food ^ Monteiro *et al.* , 2019 ^ . 

 The Nova classification was disruptive to the paradigm in the science of nutrition, and it was incorporated into a growing number of epidemiological studies. However, a common challenge for the Nova application in these studies was the use of food consumption data collected from instruments that were unable to capture differences in industrial processing. In them, for example, it was possible for all vegetable soups to be considered similar, regardless of whether they were homemade, canned, or industrially dehydrated. In this way, they could induce biases in the estimation of food groups consumption according to the Nova classification and, consequently, in identification of its determinants and consequences ^ Touvier *et al.* , 2023 ^^,^^Martinez-Steele *et al.* , 2023 ^ . 

 To overcome part of this limitation, a team from Nupens/USP developed and validated three new self-administered electronic instruments to assess food consumption according to classification: the screener of unprocessed or minimally processed whole plant foods and of ultra-processed food consumption (Screener-Nova) ^ Costa *et al.* , 2021a ^^,^^ Costa *et al.* , 2023 ^ ; the 24h dietary recall-Nova (R24h-Nova) ^ Neri *et al.* , 2023 ^ ; and the Food Frequency Questionnaire-Nova (FFQ-Nova) ^ Frade *et al.* , 2024 ^ . 

Despite the growing interest of researchers in these new instruments, there was still no online platform that made them available. This required each researcher to carry out their own programming, posing considerable obstacles—such as time, costs, and technological resources—that made the instruments inaccessible to a significant portion of the scientific community. The QuestNova platform emerged as a response to this urgent need. The purpose of this article is to describe the features, development, and functionalities of this platform.

## QUESTNOVA PLATFORM: CHARACTERIZATION AND DEVELOPMENT

QuestNova is an online platform for researchers to access, free of charge, the three instruments for collecting food consumption: the Screener-Nova, the FFQ-Nova, and the 24hR-Nova. On the platform, the researcher can select the instrument of interest and send its link to the participants of their research, who will answer it autonomously, without the presence of an interviewer. Databases containing relevant indicators for the feeding assessment according to the level of industrial processing are automatically generated from the answers, which spare researchers from the stages of raw data processing and indicators estimation.

 The platform was developed by two specialists in information technology, with the support of a team from Nupens/USP, which partially comprises researchers who also participated in the development of the instruments for assessing food consumption. The platform was developed in stages, with all the features of each step being thoroughly tested by multiple team members before moving on to the next. The tool stands out for its intuitive interface and responsive design, which allows access both via computer and by cell phone (IOS or Android) and tablet. It can be accessed at https://questnova.com.br/ . 

A crucial aspect of QuestNova is its commitment to the confidentiality and security of participants’ data. No information is stored internally on the platform; on the contrary, the data is transmitted directly to a Google Drive account provided by the researcher, thus ensuring the privacy of the collected data.

## INSTRUMENTS TO COLLECT FOOD CONSUMPTION DATA AND INDICATORS FOR DIET QUALITY ASSESSMENT

The three instruments were developed based on data on individual food consumption of the Brazilian population described in the Food Intake Surveys.

### Screener-Nova

 The Screener-Nova is a short instrument for assessing unprocessed or minimally processed whole plant foods, and ultra-processed food consumption. The participant must select the foods consumed on the previous day from a list of 33 items from the first group and 23 from the second (without informing quantities). The average time to complete the instrument is only 2 minutes. The answers result in two separate scores for each set of foods, which are calculated by simply adding one point for each consumed food and made available in the QuestNova database. A validation study revealed that each of the scores was strongly associated with the energy participation of the corresponding group, obtained from a R24h ^ Costa *et al.* , 2021a ^^,^^ Costa *et al.* , 2023 ^ . 

### R24h-Nova

 The R24h-Nova is a closed instrument that records the consumption of all foods from the previous day. The instrument comprises 57 “yes” or “no” key questions, about the consumption of key foods. Affirmative answers trigger a series of additional questions about the type and quantity consumed, method of preparation, additional items, and details to distinguish foods by processing degree. Completing the instrument takes about 15 minutes. The validation study demonstrated a moderate to good correlation between the mean estimates obtained by the R24h-Nova and those obtained by a R24h conducted by an interviewer, as well as a substantial to almost perfect agreement for the R24h-Nova ability to classify individuals into quantiles of energy participation according to the Nova groups ^ Neri *et al.* , 2023 ^ . After the validation study, adjustments to the instrument were made to improve the performance in estimating items that presented lower agreement (e.g., improvement in the question about adding olive oil to ready-to-eat foods). 

### FFQ-Nova

 The FFQ-Nova is an instrument that assesses habitual food consumption in the last 12 months. Comprising a list of 95 foods classified according to the industrial processing characteristics, the questionnaire investigates the frequency and usual amount (portions) of consumption of each item, requiring about 20 minutes to complete. A validation study described that the mean estimates of caloric participation of the Nova groups obtained by the FFQ-Nova were moderately correlated with those obtained from the mean of two R24h-Nova, while substantial agreement was observed in the classification of individuals into quantiles of energy participation of all Nova groups ^ Frade *et al.* , 2024 ^ . Considering these results, minor adjustments to the instrument were made to improve the estimation of food groups with moderate correlations (e.g., adjustment in the reference portion and the number of fruits in the questionnaire). 

 In the databases generated by QuestNova for the FFQ-Nova and R24h-Nova, the total energy consumed and the indicators (in grams, energy, and percentages of participation) of each of the groups and subgroups of the Nova classification are made available for each individual. For these calculations, recipes and nutritional composition data from the Brazilian Food Composition Table 7.0 (TBCA 7.0) are used ^Brazilian Table of Food Composition (TBCA), 2023^ , or other sources, such as the USDA table ^US Department of Agriculture, 2004^ , in the exceptional case of lack of information. 

## STEP-BY-STEP INSTRUCTIONS FOR USING THE QUESTNOVA PLATFORM

We present simplified step-by-step instructions with the necessary procedures for researchers to use the QuestNova platform (Figure 1). A complete and detailed manual is available on the platform itself.

Researcher registration:

- The researcher must register on the platform, providing personal information and a brief description of the use purpose.
- It is necessary that the registered email is linked to a Google Drive account to save and synchronize the data.

Association with Google Drive:

- After registering, the researcher must associate their account on the QuestNova platform with their Google Drive account (via an indicated spot on the interface).
- This step is essential for saving and synchronizing the collected data to the researcher’s Google Drive.

Survey Registration:

- By completing the Google Drive registration and association, the researcher can register their research on the platform.
- The researcher must fill in information such as the research title and objective, an estimate of the number of participants, and choose the instrument of use.
- It is possible to register as many studies as desired.

Sending the survey link to participants:

- After the research is registered, a unique link is generated, which must be sent by the researcher to the study participants, so that they respond to the instrument.
- Participants must enter a valid email address and their date of birth before answering the food intake assessment instrument, which are the primary key to identifying the participants and, therefore, for enabling the researcher to add the participant’s answers to the other research databases.

Database access:

- Collected data is synchronized in the researcher’s Google Drive.
- A folder named QuestNova is generated, containing the survey database in Google Sheets format.
- The database includes information on the participants’ food consumption according to the Nova classification.

## FINAL CONSIDERATIONS

This manuscript presented the QuestNova platform, which represents a technological innovation for the food consumption assessment as proposed by Nova food classification. The platform simplifies data collection and analysis, sparing researchers time and technological resources.

 The R24h-Nova and the Screener-Nova are periodically applied in the Nutrinet Brazil study ^ Werneck *et al.* , 2024 ^^-^^ Gabe *et al.* , 2023 ^ . Some examples of studies already carried out with QuestNova include one that showed an association between the caloric participation of ultra-processed foods (estimated by R24h-Nova) and the risk of depression ^ Werneck *et al.* , 2024 ^ , and another that described the relation between the scores of ultra-processed foods and unprocessed or minimally processed whole plant foods (estimated by Screener-Nova) and excessive weight gain ^ Santos *et al.* , 2023 ^ . In addition, a initial version of Screener-Nova was incorporated into 2018 VIGITEL, the 2019 *Pesquisa Nacional de Saúde* (Brazilian National Survey of Health), and the 2019 *Pesquisa Nacional de Saúde Escolar (* Brazilian National Survey of School Health) ^ Costa *et al.* , 2021b ^^,^^ Costa *et al.* , 2022 ^ . 

The availability of QuestNova democratizes access to these instruments, enhancing Brazilian studies on the impact of food processing on health. In addition, QuestNova can pose a model for other countries to develop similar platforms for incorporating instruments that assess food consumption according to Nova. Future updates may include new instruments or indicators of food quality, further strengthening their usefulness and relevance to nutritional epidemiology.
